# Color responses of the human lateral geniculate nucleus: selective amplification of S-cone signals between the lateral geniculate nucleno and primary visual cortex measured with high-field fMRI

**DOI:** 10.1111/j.1460-9568.2008.06476.x

**Published:** 2008-11

**Authors:** Kathy T Mullen, Serge O Dumoulin, Robert F Hess

**Affiliations:** 1 McGill Vision Research, Department of Ophthalmology, McGill UniversityMontreal, QC, Canada; 2 School of Optometry, Queensland University of TechnologyBrisbane, Qld, Australia

**Keywords:** color, fMRI, LGN, S-cone, V1, visual cortex

## Abstract

The lateral geniculate nucleus (LGN) is the primary thalamic nucleus that relays visual information from the retina to the primary visual cortex (V1) and has been extensively studied in non-human primates. A key feature of the LGN is the segregation of retinal inputs into different cellular layers characterized by their differential responses to red-green (RG) color (L/M opponent), blue-yellow (BY) color (S-cone opponent) and achromatic (Ach) contrast. In this study we use high-field functional magnetic resonance imaging (4 tesla, 3.6 × 3.6 × 3 mm^3^) to record simultaneously the responses of the human LGN and V1 to chromatic and Ach contrast to investigate the LGN responses to color, and how these are modified as information transfers between LGN and cortex. We find that the LGN has a robust response to RG color contrast, equal to or greater than the Ach response, but a significantly poorer sensitivity to BY contrast. In V1 at low temporal rates (2 Hz), however, the sensitivity of the BY color pathway is selectively enhanced, rising in relation to the RG and Ach responses. We find that this effect generalizes across different stimulus contrasts and spatial stimuli (1-d and 2-d patterns), but is selective for temporal frequency, as it is not found for stimuli at 8 Hz. While the mechanism of this cortical enhancement of BY color vision and its dynamic component is unknown, its role may be to compensate for a weak BY signal originating from the sparse distribution of neurons in the retina and LGN.

## Introduction

The lateral geniculate nucleus (LGN) is the primary thalamic nucleus for the neural pathway linking the retina to the primary visual cortex (V1). Its anatomy and neural properties have been extensively studied in non-human primates. One key feature of the LGN is the segregation of retinal inputs into different layers: the retinal parvocellular (P)-cells project to dorsal layers 3–6, whereas the magnocellular (M)-cell population projects to ventral layers 1 and 2, with further cell types forming the koniocellular layers of the intra-laminar regions. The neurons in these different layers have distinct neurophysiological and anatomical properties, and hence provide the basis for a functional segregation based on chromatic and spatio-temporal properties. In non-human primates, the largest neuronal population in the LGN is the P-cells; these are thought to form the basis of red-green (RG) color vision because they have L/M-cone opponency, high sensitivity to RG color contrast (Derrington *et al.*, 1984; Lee *et al.*, 1990), and lesions of the P-cell layers produce a dramatic reduction in color sensitivity (Merigan *et al.*, 1991). P-cells also respond to achromatic (Ach) contrast with good spatial resolution, low contrast gain and sustained temporal responses. M-cells are less numerous, primarily achromatic with high contrast gain maintained at high temporal frequencies (Kaplan & Shapley, 1982; Derrington & Lennie, 1984; Lee *et al.*, 1990; Solomon *et al.*, 1999). S-cone opponency, the basis of blue-yellow (BY) color vision, is carried from retina to cortex by sparse, specialized neurons mainly found in the koniocellular layers of the LGN (Martin *et al.*, 1997; Chatterjee & Callaway, 2003; Dacey & Packer, 2003).

LGN imaging provides a unique opportunity because at this thalamic stage the responses of the different chromatic signals are separated whereas at the cortical stage they become merged. Access to the LGN with functional magnetic resonance imaging (fMRI), however, has been constrained by the technical limitations imposed by its small size and the low signal strength. The location of the human LGN has been defined with high-field fMRI, but our knowledge of its functional properties remains relatively rudimentary and is so far based only on responses to Ach stimuli (Chen *et al.*, 1998a, 1998b, 1999; Fujita *et al.*, 2001; Kastner *et al.*, 2004; Schneider *et al.*, 2004), despite its large population of color-sensitive neurons. Here we report for the first time the chromatic response properties of the human LGN. Our first aim is to compare the responses of the two color pathways of the human LGN (L/M- and S-cone opponent) and the achromatic response to determine their respective contrast sensitivities based on the cone inputs. Our second aim is to understand how these responses are modified as information transfers between LGN and V1. We use an experimental design in which we acquire relative responses to the three stimulus types within one scan for comparison between LGN and V1. This approach accurately reveals relative gain changes between LGN and V1, and provides an understanding of how chromatic and Ach contrast is modified between LGN and cortex. To test the generality of our results we use two sets of stimulus contrasts, two temporal frequencies and stimuli with two different orientation compositions.

## Materials and methods

### Subjects

Eight healthy observers were used as subjects (four female, mean age 41 years, age range: 31–54 years), five of whom were naive to the purpose of the study. The subjects were instructed to maintain fixation on the provided fixation-point and trained prior to the scanning sessions to familiarize them with the task. All observers had normal or corrected-to-normal visual acuity. No participant had a history of psychiatric or neurological disorder, head trauma, or substance abuse. Informed written consent was gained from all participants prior to the commencement of the study. The study was conducted within the constraints of the ethical clearance from the Medical Research Ethics Committee of the University of Queensland for MRI experiments on humans at the Centre for Magnetic Resonance, and conforms with the Code of Ethics of the World Medical Association (Declaration of Helsinki).

### Visual stimuli

**Fig. 1:**
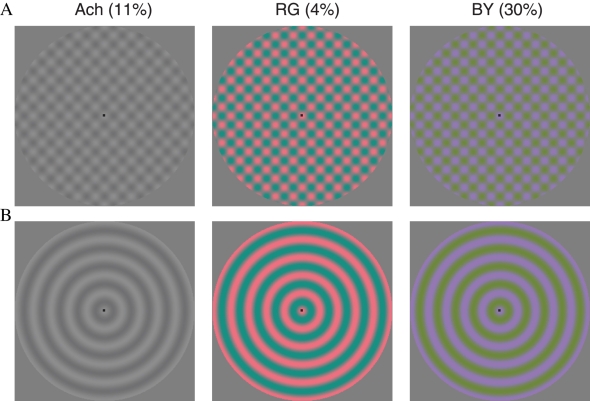
Examples of the sinewave ring and sinewave check stimuli that selectively activate the L/M-cone opponent (red-green, RG), S-cone opponent (blue-yellow, BY) or achromatic (Ach) visual mechanisms. Stimuli (0.5 cycles per degree) were sinusoidally modulated at either 2 Hz or 8 Hz. Different cone contrast sets were used in different experiments. The contrast set shown above is for relatively high-contrast checks (A) and rings (B) temporally modulated at 2 Hz, with the contrast of each stimulus threshold-scaled to 25 times detection threshold [cone contrasts of 11% (Ach), 4% (RG) and 30% (BY)]. See Materials and methods for other contrasts sets.

Stimuli were radial sinewave gratings or Cartesian sinewave checkerboards (both 0.5 cycles/degree) whose contrast phase reversed at 2 Hz or 8 Hz (see Fig. 1 for examples). All stimuli were presented in a temporal Gaussian contrast envelope (sigma = 125 ms). Three different stimulus types were used: RG, BY and Ach, which isolate L/M-cone opponent, the S-cone opponent and the Ach (luminance) post-receptoral mechanisms, respectively. [We use the color terms ‘red-green’ (RG) and ‘blue-yellow’ (BY) to refer to the stimuli that activate L/M-cone opponent, and S/(L+M)-cone opponent mechanisms, respectively. These *cone* opponent processes when activated selectively by the cardinal stimuli do not give rise to the unique color sensations of red, green, blue or yellow, and so should not be confused with *color* opponent processes]. The contrasts of the stimuli were matched either in cone contrast or in multiples of detection threshold, as described below. The stimuli were viewed as 16 degrees (full width) by approximately 12 degrees (full height), as stimulus height was limited top and bottom by the subject’s placement in the magnet bore. A small fixation dot was present in the center of the stimulus. A spatially narrow band stimulus at a relatively low spatial frequency was used to avoid artifacts generated by chromatic aberration in the chromatic stimuli (Bradley *et al.*, 1992; Cottaris, 2003), and spatial frequency was not varied experimentally due to this constraint. Radial ring (1-d in polar coordinates) and check (2-d) stimuli were used to vary the orientation bandwidth of the stimuli in order to control for the possible effect on the fMRI response of neuronal selectivities for orientation, end-stopping or other contextual effects in the cortex.

### Chromatic representation

The stimulus chromaticity was defined using a three-dimensional cone contrast space in which each axis represents the quantal catch of the L-, M- and S-cone types normalized with respect to the white background (i.e. cone contrast). Stimulus chromaticity is given by the vector direction and contrast by vector length within the cone contrast space. Three cardinal stimuli (RG, BY and Ach) were determined within this space to isolate each of the three different post-receptoral mechanisms, respectively. (A cardinal stimulus isolates one post-receptoral mechanism and is invisible to the other two, and hence is defined as the direction in cone contrast space orthogonal to the vector directions representing the other two post-receptoral mechanisms; Cole *et al.*, 1993). We selected our three cardinal stimuli from a knowledge of the cone weights of the three post-receptoral mechanisms provided by earlier studies (Cole *et al.*, 1993; Sankeralli & Mullen, 1996, 1997), and they have the following directions in the cone contrast space: the Ach stimulus activates L-, M- and S-cones equally (weights of 1, 1, 1, respectively), the BY stimulus activates S-cones only (weights of 0, 0, 1) and the isoluminant RG stimulus activates L- and M-cones opponently in proportions determined by the isoluminant point and has no S-cone activation (weights of 1, –*a*, 0). The ratio of L- to M-cone weights for RG isoluminance (value of *a*, above) was determined individually for each temporal condition for three of our eight subjects (KTM, SD, RFH) using a minimum motion method, and the average of these three isoluminant points was used for the remaining five subjects. Detection thresholds for the RG, BY and Ach stimuli (0.5 cycles per degree, radius = 10 degrees) were measured individually using a temporal 2AFC staircase procedure for the same three subjects (based on the mean of three–four staircases per condition), and the averages of these detection thresholds were used for the remaining five subjects, as given previously (Mullen *et al.*, 2007).

### Apparatus and calibrations

For all fMRI experiments, the visual stimuli were generated using psychtoolbox software (Brainard, 1997; Pelli, 1997) on a Macintosh G3 iBook and displayed on a white screen using a LCD projector (InFocus LP250, resolution 1024 × 768, frame rate = 80 Hz, mean luminance = 30 cdm^−2^). The screen was placed 2.7 m from the subject. For the psychophysical experiments used to determine detection threshold and isoluminance, stimuli were generated using a VSG 2/5 graphics board with 15 bits contrast resolution (Cambridge Research Systems, Rochester, England) housed in a Pentium PC computer and displayed on a CRT monitor (Diamond Pro 2030). Both projection and CRT displays were calibrated in the same way. The red, green and blue spectral emissions were measured using a PhotoResearch PR-650-PC SpectraScan (Chatsworth, CA, USA), and the Smith & Pokorny fundamentals (Smith & Pokorny, 1975) were used for the spectral absorptions of the L-, M- and S-cones. From these data, a linear transform was calculated to specify the phosphor contrasts required for given cone contrasts (Cole & Hine, 1992). Both displays were gamma corrected in software with lookup tables. The cone contrast gamut is most limited in the RG direction, with an upper cone contrast limit for the projection system of 5.5% for the RG stimuli, depending precisely on the calibration data and the projector settings.

### Experimental protocols and stimulus contrasts

Four different stimulus conditions were used: Ach, RG, BY stimuli, and a mean luminance (blank) condition in which only the fixation stimulus appeared. In the fixation condition, a white ring surrounded the small black fixation spot. Stimuli were presented time-locked to the acquisition of fMRI time-frames, i.e. every 3 s. To control for attention, which can affect both LGN (O’Connor *et al.*, 2002) and cortex (Brefczynski & DeYoe, 1999; Gandhi *et al.*, 1999; Somers *et al.*, 1999), subjects continuously performed a two-interval forced-choice contrast-discrimination task, in which a given presentation consisted of two intervals, both displaying stimuli from the same condition but with a small near-threshold contrast difference between them. The subject indicated which interval contained the higher contrast stimulus. The contrast difference ranged between ± 10 and ± 20% of the mean contrast for each stimulus type, and was selected based on psychophysical measurements on three subjects prior to scanning. The same contrast increments were given to all subjects. Our results showed an overall average discrimination of 82%, with no significant differences in performance for each condition indicating that attention was well controlled.

Each stimulus was presented within a 500-ms time-window in a temporal Gaussian contrast envelope (sigma = 125 ms), with an inter-stimulus interval of 500 ms. In the remaining 1.5 s the subjects’ responses were recorded using an MR-compatible computer mouse. During the mean luminance (blank) condition an identical contrast discrimination task was performed for the fixation stimulus. The four stimulus types were presented in a counter-balanced block design (six presentations per block, duration = 18s). Each block was repeated 10 times giving a total of 240 presentations per scan, i.e. 12 min/scan. All results are based on data from two scans per experiment (480 presentations, 24 min).

Experiments were performed using two different sets of stimulus contrasts and repeated for two temporal conditions (2 and 8 Hz). In the first contrast set, stimuli were presented at similar cone contrasts in order to match stimuli in terms of their respective cone responses. Due to differences in contrast sensitivity for Ach, RG and BY stimuli, these have different visibilities, with RG the most visible and BY the least. Cone contrasts used for the 2 Hz condition were 6.5% (Ach), 5% (RG), 6.5% (BY), and for the 8 Hz condition each stimulus had a cone contrast of 4.5%. The cone contrasts at 2 Hz were not exactly matched due to the 8-bit hardware limitations of the fMRI projection system, a limitation that was overcome for the 8 Hz condition; however, this difference is perceptually very small being very close to the contrast increment detection threshold. These cone contrast values were set close to the maximum limit of the cone contrast gamut of the display device for the RG color direction as this is the color direction that has the most limited cone contrast range. In the second contrast set (Fig. 1), the stimulus contrasts were presented at equivalent multiples of their respective detection thresholds. A high multiple of threshold was used in order to maximize contrast and BOLD (blood oxygen level-dependent) signal strength. For 2 Hz presentations, the cone contrasts of these stimuli were presented at 25 times their respective detection threshold and were: 11% (Ach), 4% (RG) and 30% (BY). At 8 Hz, contrasts were set to eight times their respective detection thresholds and were 2.3% (Ach), 4.5% (RG) and 20.5% (BY). All other conditions were the same in the two experiments. At 2 Hz, experiments were performed using both contrast sets for ring and check stimuli. At 8 Hz, only ring stimuli were used.

### Magnetic resonance imaging

The magnetic resonance images were acquired using a 4T Bruker MedSpec system at the Centre for Magnetic Resonance, Brisbane, Australia. A transverse electromagnetic head coil was used for radiofrequency transmission and reception (Vaughan *et al.*, 2002). For the fMRI studies, 241 T2*-weighted gradient-echo echoplanar images depicting BOLD contrast (Ogawa *et al.*, 1990) were acquired in each of 36 planes with TE 30 ms, TR 3000 ms, in-plane resolution 3.6 mm and slice thickness 3 mm (0.6 mm gap). The slices were taken parallel to the calcarine sulcus, and covered the entire occipital and parietal lobes and large dorsal-posterior parts of the temporal and frontal lobes. Two–three fMRI scans were performed in each session. Head movement was limited by foam padding within the head coil. In the same session, a high-resolution 3D T1 image was acquired using an MP-RAGE sequence with TI 1500 ms, TR 2500 ms, TE 3.83 ms, and a resolution of 0.9 mm^3^. Identification of the early visual cortical areas, including V1, was performed in separate sessions with identical parameters except for the number of time-frames (128), number of fMRI scans (1–4) and slice orientation (orthogonal to the calcarine for the retinotopic mapping experiments).

### Preprocessing of MR images

The anatomical MRI scans were corrected for intensity non-uniformity (Sled *et al.*, 1998) and automatically registered (Collins *et al.*, 1994) in a stereotaxic space (Talairach & Tournoux, 1988). The initial two time-frames of each functional run were discarded due to start-up magnetization transients in the data. All remaining time-frames were blurred with an isotropic 3D Gaussian kernel (full-width-half-maximum = 4 mm) to attenuate high-frequency noise. The functional scans were corrected for subject motion within and between fMRI scans (Collins *et al.*, 1994).

### Identification of V1 and LGN

The retinotopic mapping data were analysed using volumetric phase-encoded retinotopic mapping (Dumoulin *et al.*, 2003) as previously described (Mullen *et al.*, 2007). By combining eccentricity and polar-angle phase-maps (Engel *et al.*, 1994) with the anatomical MRI, the visual field signs could be segmented. V1 was identified as a large mirror-image representation of the visual field in and around the calcarine sulcus.

**Fig. 2:**
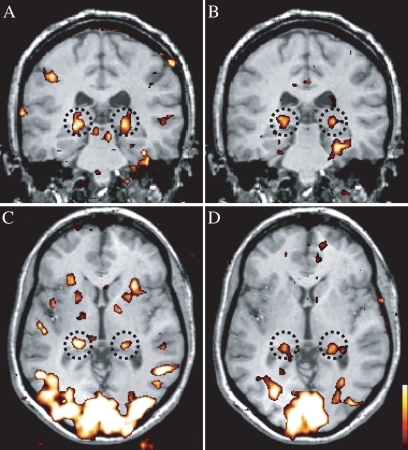
*t*-statistical maps of one subject (JS) showing a coronal (A and B) and axial view (C and D) of the left and right hemispheres in stereotaxic space (Talairach & Tournoux, 1988; Collins *et al.*, 1994). The dashed circles highlight the LGNs (for coordinates see Table 1). (A and C) Results for LGN localizing stimulus. *t*-values indicate responses to this stimulus minus a blank condition with small fixation dot. (B and D) Results for experimental stimuli. *t*-values indicate responses to all experimental stimuli (both contrast sets) minus the fixation condition.

We identified LGN based upon anatomy and two kinds of functional localizers (Fig. 2). In both cases the LGN was defined as a stimulus-responsive region in the appropriate anatomical location (Kastner *et al.*, 2004). The first functional localizer was done in a separate session using a high-contrast checkerboard stimulus with both chromatic and Ach contrast flickering (16 Hz) with both AC and DC modulation compared with a blank, black screen with a small dim fixation dot (Fig. 2A and C). The second localizer was based upon a statistical comparison of all experimental stimuli vs. fixation (Fig. 2B and D). LGN localization using these two kinds of stimuli is similar, and slight variations in the position and size of the LGN using either stimulus can be attributed to noise, registration errors and differences in stimulus layout and content. We chose to define the LGN based upon the experimental stimuli because that definition minimizes the effect of registration errors and stimulus differences when analysing the experimental stimuli. Note that this definition of the LGN compares all stimuli vs. fixation and therefore does not affect comparisons between different stimuli. The coordinates in Talairach space (Talairach & Tournoux, 1988; Collins *et al.*, 1994) of the LGNs for each subject are given in Table 1, and are similar to those previously reported (Kastner *et al.*, 2004).

**Table 1:** The LGN coordinates (mm) and volumes located in the eight subjects in stereotaxic space (Collins *et al*., 1994; Talairach & Tournoux, 1988)

	Left				Right			
	*x*	*y*	*z*	Volume (mm^3^)	*x*	*y*	*z*	Volume (mm^3^)
DA	−21	−32	0	432	18	−34	−2	304
GD	−22	−28	−6	544	22	−30	0	872
JS	−24	−30	−4	1664	22	−30	−6	1104
KLM	−22	−32	−4	552	22	−30	−4	224
KTM	−24	−24	−8	296	22	−28	−2	208
MB	−18	−28	−4	264	20	−32	−4	232
RH	−22	−26	−2	176	24	−28	−2	440
SD	−22	−35	−2	256	29	−30	−4	144
Avg	−22	−29	−4	523	22	−30	−3	441

### Statistical analysis

The fMRI data were analysed using software developed by Worsley *et al.* (2002). This statistical analysis is based on a linear model with correlated errors. The different stimulus presentations were entered into a ‘design matrix’. The design matrix of the linear model was convolved with a hemodynamic response function (Friston *et al.*, 1998; Glover, 1999). Temporal drift was removed by adding a cubic spline in the frame times to the design matrix (one covariate per 2 min of scan time), and spatial drift was removed by adding a covariate in the whole volume average. At each voxel, the autocorrelation parameter was estimated from the least-squares residuals using the Yule–Walker equations, after a bias correction for correlations induced by the linear model. Runs, sessions and subjects were combined using a linear model with fixed effects and standard deviations taken from the previous analysis on individual runs. A random effects analysis was performed by first estimating the ratio of the random effects variance to the fixed effects variance, and then regularizing this ratio with a Gaussian filter (10 mm fwhm – note that this filter does not actually smooth the data). The variance of the effect was then estimated by the smoothed ratio multiplied by the fixed effects variance to achieve higher degrees of freedom. The resulting *T*-statistical images were thresholded for peaks and cluster sizes using random field theory (Worsley *et al.*, 1996).

The volume of interest (VOI) analysis of V1 and the LGN was done in an identical fashion. Prior to the statistical analysis, the fMRI signal fluctuations are converted to % BOLD signal change relative to the mean signal intensity level for the scan on a per voxel basis. For example, −50 and 100% signal change correspond to a half and double the mean signal intensity of the fMRI signal, respectively, and the 0% on the figures is set by the mean signal intensity of the scan. Time-series of voxels within a VOI (left and right hemispheres) were averaged together, with exclusion of voxels displaying artifacts. Voxels with artifacts were identified by their unusually large intensity variations in their time-series, i.e. voxels with a standard deviation larger than 19 (although the final results were stable across a range of thresholds). The VOI analysis was performed on each subject’s areas and subsequently averaged across subjects. Because the time-series were converted to % BOLD signal change the effect size of the linear model (β) is also in percent signal change. The effect sizes and their standard deviations relative to the overall mean of the time-series are plotted in the data figures.

## Results

**Fig. 3:**
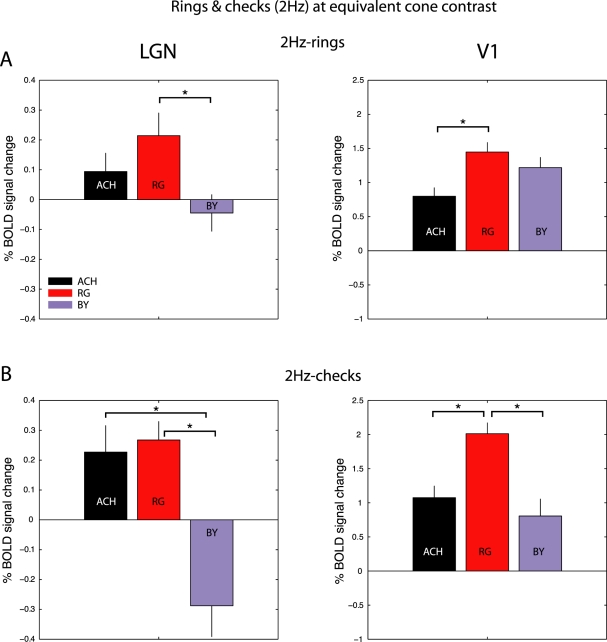
Results of VOI analyses for the lateral geniculate nucleus (LGN) and primary visual cortex (V1) for red-green (RG), blue-yellow (BY) and achromatic (Ach) stimuli matched in cone contrast and presented with 2 Hz sinusoidal temporal modulation. VOI analyses were performed for each subject individually and subsequently averaged across subjects. The ordinate shows the average percentage blood oxygen level-dependent (BOLD) signal change and SD for each condition. % signal change is calculated relative to the mean signal intensity level averaged for all conditions across the scan. Histograms are color coded according to the stimulus type: red for RG isoluminant stimuli that selectively activate L/M-cone opponent pathways; blue for BY isoluminant stimuli that selectively activate the S-cone opponent pathways; and black for Ach stimuli that fail to activate the two chromatic mechanisms above. Left panels show data for the LGN and right panels for V1 obtained on the same subjects in the same scans. Stimuli were presented at 2 Hz sinusoidal temporal modulation and were sinewave rings in (A) with results averaged across eight subjects (16 LGNs), and sinewave checks in (B) averaged across five subjects (10 LGNs). Full statistical comparisons are given in Table 2, but significant effects with a Bonferroni correction for multiple *t*-test comparisons (*n*=2, based on the two areas examined) are marked with an asterisk and are as follows: in (A) for the LGN, RG > BY; in (A) for V1, RG > Ach; in (B) for the LGN, RG > BY, Ach > BY; in (B) for V1, RG > Ach, RG > BY. All responses are significantly greater than the fixation condition.

**Fig. 4:**
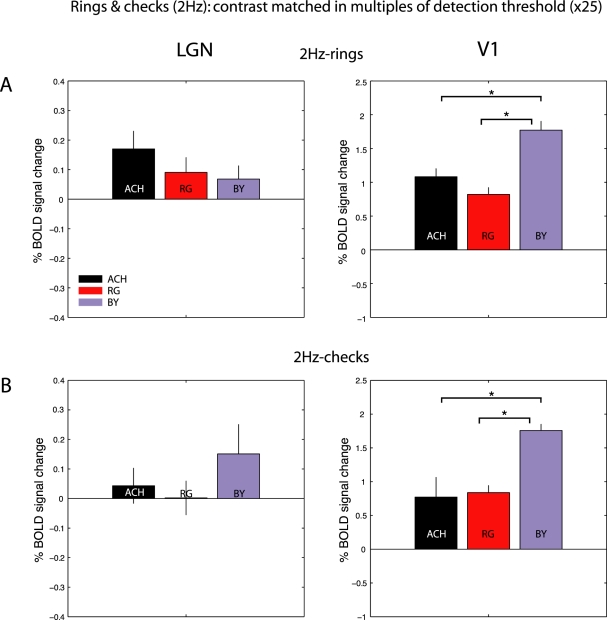
Results of VOI analyses for the lateral geniculate nucleus (LGN) and primary visual cortex (V1) for stimuli presented at high suprathreshold contrasts (25 × their respective detection thresholds) at 2 Hz sinusoidal temporal modulation. Plot details are the same as for Fig. 3. Stimuli were sinewave rings (A) with results averaged across eight subjects (16 LGNs) or sinewave checks (B) averaged across five subjects (10 LGNs). Full statistical comparisons are given in Table 2, but significant effects with a Bonferroni correction for multiple *t*-test comparisons (*n*=2, based on the two areas examined) are marked with an asterisk and are as follows: in (A and B) for V1, BY > Ach and BY > RG. All responses are significantly greater than the fixation condition. Ach, achromatic; BOLD, blood oxygen level-dependent; BY, blue-yellow; RG, red-green.

**Fig. 5:**
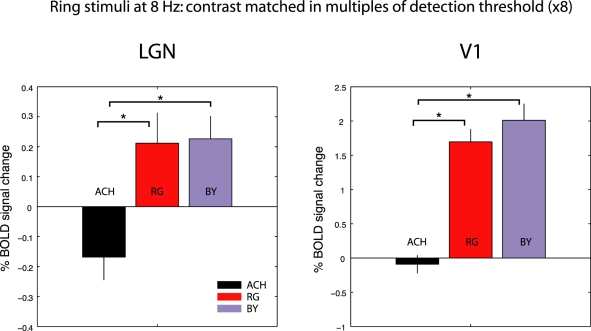
Results of VOI analyses for the lateral geniculate nucleus (LGN) and primary visual cortex (V1) for stimuli presented at 8 Hz sinusoidal temporal modulation and matched at suprathreshold contrasts (8 × their respective detection thresholds). Plot details are the same as for Fig. 3. Stimuli were sinewave rings with results averaged across seven subjects (14 LGNs). Full statistical comparisons are given in Table 2, but significant effects with a Bonferroni correction for multiple *t*-test comparisons (*n*=2, based on the two areas examined) are marked with an asterisk and are as follows: for the LGN and V1, RG > Ach, BY > Ach. All responses except the Ach in the LGN are significantly greater than the fixation condition. Ach, achromatic; BOLD, blood oxygen level-dependent; BY, blue-yellow; RG, red-green.

In Figs 3–5 we show the results of the VOI analyses for the isoluminant RG, BY and Ach stimulus conditions in the LGN (left panels) and V1 (right panels). BOLD signal change (% change relative to the mean signal intensity level averaged across the scan) is calculated and the plots show the averages across subjects. In Fig. 3 the three stimulus types (RG, BY and Ach) are matched in cone contrast (see Materials and methods), and stimuli are sinewave rings (Fig. 3A) or a sinewave Cartesian checkerboard pattern (Fig. 3B) sinusoidally phase reversed at 2 Hz with results for LGN shown in the left panels and for V1 in the right panels. Equating stimuli directly in cone contrast ensures that the neural activity for each stimulus is equivalent at the level of the cone responses, and hence the BOLD responses obtained indicate relative sensitivity of the LGN and V1 for each of the different contrast types. Results show that for both the ring and check stimuli the greatest LGN activation is found for RG contrast, which is significantly greater than the BY response, with the Ach response falling in between (see Fig. 3 and Table 2 for statistical data). Thus, the results indicate that for 2 Hz stimuli the LGN has a significantly greater activation of the L/M-cone opponent than the S-cone opponent pathway.

**Table 2:** The *t-* and *P-*values for the VOI analyses plotted in Figs 3–5 and for Supplementary Fig. S1

	Stimulus TFContrast set											
	RINGS 2 Hz CC, Fig. 3		CHECKS 2 Hz CC, Fig. 3		RINGS 2 Hz MDT, Fig. 4		CHECKS 2 Hz MDT, Fig.4		RINGS 8 Hz MDT, Fig.5		RINGS 2 Hz, Fig. S1	
Brain region	LGN	V1	LGN	V1	LGN	V1	LGN	V1	LGN	V1	LGN	V1
Ach-RG												
*T*-value	−0.88	−3.99	−0.45	−5.55	0.54	1.84	0.44	−0.20	−2.40	−6.72	−1.77	−1.45
*P*-value	0.57	0.001*	0.98	0.001*	0.88	0.10	0.99	1.26	0.025*	0.001*	0.12	0.22
Ach-BY												
*T*-value	1.40	−1.65	3.42	0.79	0.73	−4.54	−0.81	−2.93	−4.38	−6.63	−0.91	−4.34
*P*-value	0.24	0.15	0.001*	0.64	0.70	0.001*	0.63	0.005*	0.001*	0.001*	0.54	0.001*
BY-RG												
*T*-value	−2.20	−1.07	−3.87	−3.18	−0.14	4.84	1.34	7.04	0.11	1.48	−0.47	3.74
*P*-value	0.042*	0.43	0.001*	0.002*	1.33	0.001*	0.27	0.001*	1.37	0.21	0.96	0.001*
Ach-Col												
*T*-value	0.24	−2.88	2.07	−1.66	0.78	−1.28	−0.43	−1.57	−4.12	−7.20	−1.78	−3.77
*P*-value	1.22	0.006*	0.058	0.15	0.65	0.30	1.00	0.17	0.001*	0.001*	0.11	0.001*

The *t-*values are for the differences in cortical responses between the different stimulus conditions shown (Col, averaged RG and BY). *Significant differences (*P*=0.05) are based on multiple *t-*test comparison between conditions with a Bonferroni correction for multiple comparisons based on the two brain areas examined (Worsley *et al.*, 1996, 2002). The sign of the *t-*value indicates which response is the greater. *P-*values of 0.001 represent a value of 0.001 or less.

MDT, multiples of detection threshold.

Results for V1 are shown in the right panels. Signal strength is much higher than in the LGN as has been shown previously for Ach stimuli (Kastner *et al.*, 2004); however, we are interested in the relative comparison of the responses across the three stimulus types. For both ring and check stimuli, V1 responds significantly better to RG than to Ach stimuli, a pattern of results that has been reported previously (Engel *et al.*, 1997; Liu & Wandell, 2005; Mullen *et al.*, 2007). V1 also shows a robust response to BY stimuli; for the ring stimuli the cortical response to the BY stimulus is similar to that for the RG and Ach stimuli, whereas in the LGN the BY response was significantly less than the RG response. For the check stimuli in V1, the BY and Ach activations are similar, whereas in the LGN the BY activation was significantly less than the Ach and RG. Thus, a key feature of the comparison of the responses between the LGN and V1 is a marked increase in the BY response relative to the Rt and Ach, which occurs for both ring and check stimuli.

We repeated these comparisons using a second set of contrasts for the stimuli. We used a high suprathreshold contrast scaled in multiples of detection threshold (25 × detection threshold) for all stimuli in order to maximize signal strength across the different chromatic conditions. In particular to achieve these suprathreshold values the contrasts of the Ach and BY stimuli were increased from their previous values. The contrast of the RG stimulus remains of a similar value to the previous set as this stimulus is already well suprathreshold due to the very low detection threshold for RG chromatic contrast. These three contrasts are the highest we can achieve while remaining within the calibrated limit of the color gamut of the display. Results are shown in the left panels of Fig. 4 for the LGN, and in the right panels for V1 for ring (A) and check (B) stimuli. LGN activations by Ach, RG and BY stimuli are similar with no significant differences between them. This is a different pattern of response to that found for stimuli at equivalent cone contrasts (Fig. 3A), and is expected on the basis of the increased contrasts of the BY and Ach stimuli in this condition. The results for V1, obtained from the same scans, show significantly greater response to the BY stimuli than to the RG and Ach. A strong BY response in V1 for stimuli matched at high suprathreshold contrasts has been reported previously (Mullen *et al.*, 2007). However, here we show that this robust BOLD response to BY in V1 is not present in the LGN, so demonstrating a relative strengthening of the response to BY contrast at the cortical level. We find similar results for both ring and check stimuli. Because our data sets were collected over an extended time period (2 years), we repeated the first experiment (2 Hz ring stimuli) on four of our original eight subjects to check on the stability of our results over time. These data are shown in Supporting information Fig. S1 and show the same pattern of results as found in Fig. 4A, with no significant differences in the response of the LGN to the three threshold-scaled stimuli but a significantly stronger response in the cortex to the BY stimulus than to the other two. Overall our results for stimuli modulated at 2 Hz show the same relative strengthening of the response of the S-cone opponent pathway between LGN and V1 for both contrast sets.

Next we extended our experiments to a higher temporal frequency of 8 Hz. For stimuli presented at matched cone contrasts (4.5%), we were unable to obtain LGN activation significantly above the fixation condition for any of the three stimuli. This may be caused by a general weakening of the LGN BOLD response at 8 Hz (Mullen *et al.*, 2008), as well as the relatively poor visibility in the case of the BY stimuli due to the cone contrast matching of the stimuli. Due to the weak LGN activations by these stimuli we have excluded them from further analysis, and we focus instead on the second contrast set in which we use threshold-scaled stimuli presented at eight times their respective detection thresholds, which allows us to increase the contrast of the BY stimuli. Because chromatic thresholds rise as temporal frequency increases (Kelly, 1983; Mullen & Boulton, 1992), the suprathreshold scaling factor of × 8 is lower than the value used in the previous experiment, but is the highest threshold-scaled contrast we could obtain for all three stimuli within the limit of the color gamut. Results are shown in Fig. 5 (with statistical values in Table 2). Activation of the LGN (left panel) indicates a robust response to both RG and BY stimuli, but a significantly smaller activation by Ach stimuli. It is important to note that, because stimuli all have different cone contrasts (due to the threshold-scaling), these activations do not directly reflect the relative sensitivities of the LGN to Ach, RG and BY contrast. V1 also shows a similar activation pattern with no significant difference between activation by RG and BY contrast, but a significantly weaker activation by the Ach stimuli. Thus, the comparison of LGN and V1 indicates no change in the relative response of the S-cone opponent pathway between LGN and cortex, contrary to our results for the 2 Hz stimuli. [At the request of one of our reviewers, we show the excluded data sets obtained for the stimuli matched in cone contrast (mentioned above) and a second data set obtained for low-contrast stimuli (4 × detection threshold) in supporting Fig. S2, although we note that the LGN responses for these conditions are not significantly greater than the fixation condition].

We think that the relatively low response to Ach contrast compared with the color stimuli at 8 Hz may be influenced by the threshold-scaling procedure. At 8 Hz psychophysical Ach detection thresholds are very low and resemble a flicker threshold, as described previously and thought to depend on M-cell responses (King-Smith & Kulikowski, 1975), an effect not found for the chromatic thresholds. When contrast is multiplied up by the threshold-scaling factor, this translates into an unequal visibility change across the stimuli, with Ach stimuli less visible than the chromatic ones, and may reflect an M-cell contrast saturation effect (Kaplan & Shapley, 1982). It is important to note, however, that this has no bearing on our comparison of the relative changes in activation by the three stimulus types between LGN and V1, which are independent of the absolute contrasts selected.

Next we apply a quantitative comparison between the relative responses of the LGN and V1 to the three different contrast types in order to test more directly how the V1 response to BY contrast changes in relation to RG and Ach contrast. The LGN signal amplitudes [*S*(LGN)_Ach,RG_] were projected onto the V1 data values *S*(V1)_Ach,RG_ for the combined Ach and RG contrast conditions using a fit of a general linear model: 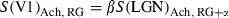
(1) where β represents the slope/gain and α represents the fMRI baseline differences between the LGN and V1 data. The projection error for the BY condition (*e*_BY_) is then calculated from this fit, 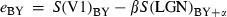
(2)

**Fig. 6:**
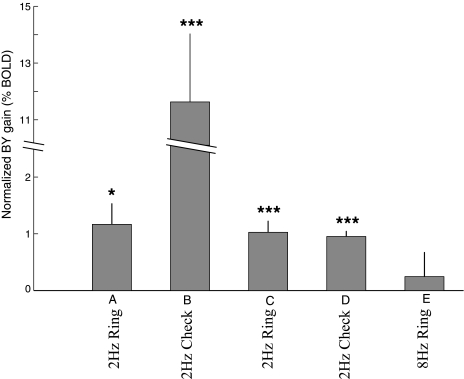
LGN to V1 gain changes for BY stimuli normalized relative to the RG and Ach gains. The BY gain is significantly higher (*P<0.01, ***P<0.0001) for 2 Hz ring (A) and check (B) stimuli presented in equal cone contrasts (see Fig. 3), 2 Hz ring (C) and check (D) stimuli presented at equal multiples of threshold (see Fig. 4). There is no difference in BY gain for ring stimuli presented at 8 Hz (E) (at equal multiples of threshold; see Fig. 5).

This projection error is plotted in Fig. 6 for the BY condition. By definition for the RG and Ach conditions the errors are zero and so are not shown. We have also computed the fit of Eqn (1) (α, β) based upon all three conditions (Ach, RG, BY) and the final results are similar, with the projection error distributed across all conditions but in opposite signs for BY as compared with Ach, RG. This method removes the effect of absolute constrast on BOLD response by normalization.

Results show that the projection error is significantly greater than 0 for all BY stimuli at 2 Hz (Fig. 6A–D). This demonstrates that the BY activation has a significant differential increase between LGN and V1, over and above the increase for RG and Ach stimuli. For the 8 Hz condition (Fig. 6E) the projection error is not significantly greater than 0, showing that the increase in BY activation between LGN and cortex is similar to that found for RG and Ach stimuli with no differential effects.

## Discussion

We have shown for the first time reliable activation of the human LGN to stimulation of the two different LGN color pathways (L/M-cone opponent and S-cone opponent), as well as to achromatic stimulation, using high-field (4T) fMRI. This has allowed us to address two key issues, one is the relative sensitivity of the human LGN to these three different types of contrast, and the other is how these signals are modified between LGN and V1.

### fMRI responses of the human LGN

In the first experiment, we used stimuli of 2 Hz presented at equivalent cone contrasts because this matches the cone inputs to the three systems, and so indicates the relative contrast sensitivity of the LGN BOLD response to the chromatic and achromatic contrasts. We find that LGN activation is significantly greater for RG than BY stimuli. This is likely to reflect, at least in part, the wide difference in the numbers of neurons activated by each stimulus. L/M-cone opponency is supported by the primate P-cells of the LGN, which respond optimally to RG contrast (Derrington *et al.*, 1984; Lee *et al.*, 1990; Solomon & Lennie, 2005) and form the large majority of the cells in the LGN (Shapley & Perry, 1986). S-cone opponency, on the other hand, is supported in the primate LGN by a much smaller group of sparse, specialized neurons (Derrington *et al.*, 1984; Reid & Shapley, 1992), reflecting the low proportion of S-cones in the retinal cone population (∼7%; Curcio *et al.*, 1991). The lower discharge rate of koniocellular (K)-cells compared with P-cells (White *et al.*, 2001), notably seen in the ‘Blue-OFF’ cells (Solomon & Lennie, 2005), may also contribute to the relatively small LGN response to BY stimuli. In the second experiment we increased the contrasts of the BY and Ach stimuli, using high-contrast threshold-scaled stimuli to compensate for their poorer contrast sensitivities, and were able to obtain similar robust and reliable responses from all three stimulus types. Results were similar for sinewave ring and check stimuli, which is not surprising given the isotropic nature of LGN neural responses. In the third experiment we investigated the effects of temporal frequency by increasing our stimulus temporal frequency to 8 Hz, which was as high as we could obtain while remaining in the visible range for color vision (e.g. 16 Hz is invisible for RG and BY stimuli). At 8 Hz for stimuli presented at equivalent cone contrasts (4.5%), we could not obtain reliable activation (significantly above the fixation condition) across the three stimulus types and so could not compare the relative contrast sensitivities, as discussed above. When the BY contrast was increased by the use of high-contrast threshold-scaled stimuli, a chromatic response was obtained for BY as well as RG contrast. This indicates that even though psychophysical color sensitivity has declined significantly at 8 Hz (Kelly, 1983), the LGN response is still robust in both L/M- and S-cone opponent pathways.

Overall the very robust activation of the human LGN by L/M-cone opponent stimulation indicates that the addition of color contrast is a powerful activator of human LGN and is an effective tool for boosting signal strength in the future investigation of the LGN by fMRI. Furthermore, now that we have demonstrated that selective activation of the LGN by S-cone opponent and L/M-cone opponent pathways can be obtained, other properties such as different aspects of the temporal or spatial characteristics of their response or their retinotopic organization can be investigated in more detail.

In this study we have focused on the chromatic responses of the LGN, and hence have used parameters within the spatio-temporal pass band of human color vision. For this reason we have used relatively low spatial frequencies with sinusoidal profiles to avoid the contribution of artifacts from chromatic aberrations (Bradley *et al.*, 1992) and to optimize color sensitivity (Mullen, 1985). Future use of higher spatial or temporal frequencies, outside the visible range of color vision, will allow a fuller investigation of the achromatic system. The achromatic stimulus potentially activates both M- and P-cells, with the relative responses depending on spatio-temporal conditions and contrast gains of these neurons in ways not yet fully understood for human vision. Relatively low Ach contrasts (< 10%) are used in Fig. 3 (experiment 1) and Fig. 5 (experiment 3), and so are likely to favor M-cell over P-cell responses in these experiments (Lee *et al.*, 1990; Sclar *et al.*, 1990). The relatively low activation by achromatic compared with chromatic contrast at 8 Hz (Fig. 5) for the threshold-scaled stimuli in both LGN and V1 may reflect a contrast saturation effect possibly operating at the level of the M-cells.

### Gain changes between human LGN and V1 for chromatic and Ach contrast

In addition to comparing the LGN responses to chromatic and achromatic stimuli, the focus of this study is to investigate how the relative thalamic responses of these three pathways are modified as information travels between LGN and cortex over a range of spatio-temporal conditions (1d, 2d stimuli, 2 Hz and 8 Hz) by comparing the relative activations of the LGN and V1 for the RG, BY and Ach stimuli. The BOLD responses of the cortex to chromatic and Ach stimuli have been reported previously, and typically show strong color responses both in V1 and in the visual areas of the ventral cortical stream, and will not be discussed further here (Engel *et al.*, 1997; Liu & Wandell, 2005; Mullen *et al.*, 2007). It is an important part of our experimental design that LGN and V1 activations are recorded simultaneously and the activations by the three stimulus types are acquired during the same scan in order to eliminate any effects of inter-scan variations. We have found that, at 2 Hz, the activation produced by stimulation of the S-cone opponent pathway is enhanced in V1 relative to the LGN, indicating a significant differential gain change in this pathway at the cortical level. This effect was significant for both spatial stimuli (ring and checks), for both contrast sets, and remained significant when repeated on four of our original eight subjects 2 years later. On the other hand, no significant effect was present at 8 Hz and relative activations for the chromatic and achromatic stimuli did not change significantly between LGN and V1.

The enhancement of the cortical activation for BY stimuli that we have found reveals the presence of selective changes in the response of the S-cone pathway between LGN and cortex that are not found in the L/M opponent or achromatic pathways. These differential changes occurring between LGN and V1 in the S-cone pathway may be either in the response gains of individual neurons, in the numbers of S-cone neurons responding (population effects) or in the spatio-temporal tuning of the S-cone neurons, or some combination of these effects. Below, we critically assess the evidence for three possible sources of these changes.

The first possibility is that individual S-cone opponent neurons may show a selective increase in contrast gain relative to their L/M counterparts, so contributing to an enhanced BY fMRI response in V1. There is some evidence for this in macaque V1 as Cottaris & De Valois (1998) have shown, based on a sample of 95 neurons over 12 monkeys that cortical S-cone opponent signals are selectively amplified by a dynamic non-linearity, possibly based on intra-cortical modulation, yielding a delayed and sluggish S-cone signal in the cortex. The slow temporal dynamics of this effect could also account for why we found no selective enhancement of the BY response at 8 Hz, both because the amplification does not occur at 8 Hz, and because there are proportionally fewer S-cone responses at 8 Hz in the cortex as neurons have become more sluggish.

This study argued that the single-cell amplification of BY contrast serves to compensate for the extreme paucity of BY neurons found at the level of the retina and LGN. While the evidence for this single-cell amplification rests on only one study of less than 100 cells in macaque, our fMRI results lend support to these neurophysiological results by revealing that in human vision there is an overall amplification of the S-cone signal at the cortical level. It is not yet possible to quantitatively assess the contribution of single-cell amplifications of the type reported above in primate, and furthermore other possible effects are also likely to be involved.

A second possible cause of the enhanced BY response is a differential expansion in the proportion of neurons receiving S-cone inputs relative to L- and M-cone inputs between LGN and V1, and there is considerable evidence for this. There is a major change in the distribution of the chromatic tuning of the color-sensitive neurons between the LGN and cortex, from a bimodal distribution representing two distinct types of color tuning in the LGN (S-cone and L/M-cone opponent) to a broader distribution that includes a wider variation in how the cones are combined into different cell types in V1 (Lennie *et al.*, 1990; De Valois *et al.*, 2000; Johnson *et al.*, 2001, 2004; Wachtler *et al.*, 2003; Solomon & Lennie, 2005; Conway & Livingstone, 2006; Horwitz *et al.*, 2007). One of the reported effects of this is to increase the proportion of S-cone responses in the total cone response as S-cones feed a broader range of neurons at the cortical level. The size of this increased S-cone contribution at the cortical level is difficult to estimate and is likely be influenced by response non-linearities (Horwitz *et al.*, 2007), although an approximate doubling of the S-cone response at the cortical level has been suggested (a rise from a 10% contribution of S-cones at the level of the LGN to near 20% in V1; De Valois *et al.*, 2000). However, it should be noted that these neurophysiological effects have been disputed (Johnson *et al.*, 2004). It cannot yet be quantitatively assessed whether an increased proportion of S-cone contributions to cortical neurons is sufficient to account for the very similar levels of cortical activation by BY, RG and Ach contrast that we find in the cortex, and other factors are also likely to contribute.

A third possible source of the enhancement effect lies in the nature of fMRI as a population response and depends on the relative numbers of neurons responding to the stimuli at the different stages of processing. The strength of the fMRI signal will depend on the interaction between the spatial tuning of all neurons and the spatial properties of the stimulus employed (Schluppeck & Engel, 2002), which determines the proportion of neurons activated as well as the strength of their response. Fundamental neural changes occur between LGN and cortex, including the addition of orientation tuning and an increase in the selectivity of tuning for spatial frequency in the cortex, as well as the presence of surround and contextual effects. Potentially all of these can differentially affect the fraction of neurons responding to the different chromatic and achromatic contrasts of our stimuli in cortex vs. LGN. For example, orientationally 1D stimuli in the LGN would activate all LGN neurons, as they are all orientationally isotropic, whereas in the cortex such stimuli will activate only the subset of the cortical neurons that have a matched orientation tuning plus any isotropic cortical neurons. Hence, for 1D stimuli, any difference in the degree of orientation selectivity between color and achromatic neurons in the cortex may influence how the BOLD response changes between LGN and cortex. For this reason we have used control stimuli that are orientationally isotropic (checks), which activate all neurons whether isotropic or narrowly tuned. The fact that the enhanced BY response is obtained for isotropic stimuli indicates that it is not produced by any differences in orientation selectivity between S-cone and L/M-cone opponent neurons or luminance stimuli at the level of V1. By the same argument, this result also controls for any differences in end-stopping or other contextual effects that might exist between the cortical S-cone and L/M-cone opponent or achromatic neurons, which might potentially produce differential activation within a neural population when spatially 1D stimuli are used.

Similar arguments may be also applied based on the spatial frequency of the test stimuli. Use of a narrow-band stimulus of relatively low spatial frequency will result in a high proportion of lowpass neurons responding, whereas only a subset of narrowly tuned neurons will respond. For example, in the LGN, cone opponent neurons are lowpass for color contrast (Derrington *et al.*, 1984). In the cortex, however, the presence of a greater proportion of lowpass spatial tuning in S-cone neuronal population compared with L/M might act to cause a differential increase in the cortical fMRI activation by BY contrast. The available neurophysiological data on this are very limited, but suggest that there are no substantial differences between the spatial tuning of the chromatic L/M- and S-cone opponent systems in macaque V1 (Johnson *et al.*, 2001, 2004), so tending to rebut this argument. This possible effect could potentially be tested directly by fMRI experiments using spatially broadband stimuli that activate neurons regardless of their spatial selectivities; however, this would involve introducing higher spatial frequencies into our stimuli, which we cannot do as higher spatial frequencies introduce chromatic aberration and luminance artifacts into the stimuli (Cottaris, 2003). In conclusion, the robust response of human V1 to S-cone activation is surprising in view of the sparse distribution of S-cone inputs estimated from primate data. Our results reveal that this effect in not present in the LGN but arises from a differential increase in BOLD activation to S-cone responses occurring between LGN and cortex.

## Abbreviations

BOLD: blood oxygen level-dependent
fMRI: functional magnetic resonance imaging
L-: M- or S-cone, long-, medium- or short-wavelength absorbing, respectively
LGN: lateral geniculate nucleus
M: magnocellular
P: parvocellular
RG: BY and Ach, red-green, blue-yellow and achromatic, respectively
V1: primary visual cortex
VOI: volume of interest

## Supporting information

Fig. S1. Plot details and stimuli are as for Fig. 4A.

Fig. S2. Plot details and stimuli are as for Fig. 5.
