# Supplementary material for: Color responses of the human lateral geniculate nucleus: selective amplification of S-cone signals between the lateral geniculate nucleno and primary visual cortex measured with high-field fMRI
Source: Eur J Neurosci. 2008 Nov;28(9):1911–23. doi: 10.1111/j.1460-9568.2008.06476.x (PMC2777261; doi:10.1111/j.1460-9568.2008.06476.x)
Supplement: Supplementary file 1 [file ejn0028-1911-SD1.doc]

**Fig. S1.** Plot details and stimuli are as for Fig. 4A. Results are averaged across 4 subjects (8 LGNs) for 2 Hz ring stimuli presented at contrasts of 25x their respective detection thresholds. Significant effects are marked with an asterisk and are: for V1 BY>Ach, BY>RG (see Table 2). All responses except the BY in the LGN are significantly greater than the fixation condition.
