# Supplementary material for: Color responses of the human lateral geniculate nucleus: selective amplification of S-cone signals between the lateral geniculate nucleno and primary visual cortex measured with high-field fMRI
Source: Eur J Neurosci. 2008 Nov;28(9):1911–23. doi: 10.1111/j.1460-9568.2008.06476.x (PMC2777261; doi:10.1111/j.1460-9568.2008.06476.x)
Supplement: Supplementary file 2 [file ejn0028-1911-SD2.doc]

**Fig. S2.** Plot details and stimuli are as for Fig. 5. Results are averaged across 7 subjects (14 LGNs) for 8 Hz ring stimuli. In A, stimuli are matched in cone contrast (4.5% each condition) and in B are presented at contrasts of 4x their respective detection thresholds (1.2% for Ach, 2.3% for RG, and 10.2% for BY). None of the responses in the LGN for either A or B are significantly above the fixation condition and so have not been included in the main analysis. (In A, the BY condition is significantly below the fixation condition, and in B, Ach is significantly below the fixation condition. We note that the fixation condition generates some BOLD activity in the LGN.) Significance measures indicate comparisons between stimulus conditions as made previously (eg. Figure 5). Trends in the data support the main analysis for 8 Hz stimuli shown in Figure 5 for stimuli matched at 8x their respective detection thresholds.
